# Biochemical characterization of a SusD-like protein involved in β-1,3-glucan utilization by an uncultured cow rumen *Bacteroides*

**DOI:** 10.1128/msphere.00278-24

**Published:** 2024-07-16

**Authors:** Xiaoqian Li, Guy Lippens, Jean-Luc Parrou, Gianluca Cioci, Jérémy Esque, Zhi Wang, Elisabeth Laville, Gabrielle Potocki-Veronese, Aurore Labourel

**Affiliations:** 1 TBI, Université de Toulouse, CNRS, INRAE, INSA, Toulouse, France; University of Wisconsin-Madison, Madison, Wisconsin, USA

**Keywords:** β-1,3-glucan, SusC/D transporter, uncultured *Bacteroides*, metagenomics, cow rumen

## Abstract

In ruminants, the rumen is a specialized stomach that is adapted to the breakdown of plant-derived complex polysaccharides through the coordinated activities of a diverse microbial community. Bacteroidota is a major phylum in this bovine rumen microbiota. They contain several clusters of genes called polysaccharide utilization loci (PULs) that encode proteins working in concert to capture, degrade, and transport polysaccharides. Despite the critical role of SusD-like proteins for efficient substrate transport, they remain largely unexplored. Here, we present the biochemical characterization of a SusD-like protein encoded by a β-glucan utilization locus from an *Escherichia coli* metagenomic clone previously isolated by functional screening of the bovine rumen microbiome. In this study, we show that clone 41O1 can grow on laminaritriose, cellotriose, and a mixture of cellobiosyl-cellobiose and glucosyl-cellotriose as sole carbon sources. Based on this, we used various *in vitro* analyses to investigate the binding ability of 41O1_SusD-like towards these oligosaccharides and the corresponding polysaccharides. We observed a clear binding affinity for β-1,6 branched β-1,3-glucans (laminarins, yeast β-glucan) and laminaritriose. Comparison of the AlphaFold2 model of 41O1_SusD-like with its closest structural homologs highlights a similar pattern of substrate recognition. In particular, three tryptophan residues are shown to be crucial for laminarin recognition. In the context of the cow rumen, we discuss the possible substrates targeted by the 41O1_PUL, such as the (1,3;1,4)-β-d-glucans present in cereal grains or the β-1,3- and (1,3;1,6)-β-d-glucans that are components of the cell wall of ruminal yeasts.

**IMPORTANCE:**

The rumen microbiota can majorly impact overall animal health, feed efficiency, and release of harmful substances into the environment. This microbiota is involved in the fermentation of organic matter to provide the host with valuable and assimilable nutrients. Bacteroidota efficiently captures, breaks down, and imports complex polysaccharides through the concerted action of proteins encoded by polysaccharide utilization loci (PULs). Within this system, SusD-like protein has proven necessary for the active internalization of the substrate. Nevertheless, the vast majority of SusD-like proteins characterized to date originate from cultured bacteria. With regard to the diversity and importance of uncultured bacteria in the rumen, further studies are required to better understand the role of polysaccharide utilization loci in ruminal polysaccharide degradation. Our detailed characterization of the 41O1_SusD-like therefore contributes to a better understanding of the carbohydrate metabolism of an uncultured *Bacteroides* from the cow rumen.

## INTRODUCTION

The rumen, a specialized compartment within the digestive system of ruminant animals, such as cows, serves as a fermentation chamber for digesting food. It facilitates the breakdown of complex plant fibers and polysaccharides through the coordinated activities of a diverse microbial community. These microorganisms produce short-chain fatty acids, microbial proteins and vitamins, which provide the nutrients that the host needs for maintenance and growth (1–3). The composition and activity of the rumen microbiota are largely determined by diet, among other factors (4), and are crucial for overall animal health, improving feed efficiency, and minimizing the release of harmful substances into the environment (5–7).

With the advent of large-scale anaerobic culture-based methods (8) and the boom in various “omics” approaches, it is now possible to map the phylogenetic diversity of the rumen microbiota. This is a multi-kingdom ecosystem containing bacteria (9), ciliate protozoa (10), methanogens (*Archaea)* (11), anaerobic fungi (12) and viruses (13). Because bacteria are active players in plant biomass degradation, many recent studies have investigated their carbohydrate-active enzyme (CAZyme) content (9, 14–18). Bacteriodota, one of the dominant phyla of the rumen microbiota, possesses a particularly vast arsenal of CAZyme genes, which are colocalized in polysaccharide utilization loci (PULs) (19). The first canonical PUL was uncovered through pioneering studies of dietary starch utilization by the human gut symbiont *Bacteroides thetaiotaomicron*. According to the “starch utilization system” (Sus) paradigm (20–22), polysaccharides are first degraded by a cell envelope-associated multiprotein system (the “Sus”) that enables the bacterium to bind and degrade carbohydrates efficiently. Derivatives of this prototypic system (“Sus-like systems”) are highly represented in the genome of many other saccharolytic Bacteroidota. Next, SusD-like substrate-binding proteins, sometimes together with the other surface glycan-binding proteins (SGBPs), selectively recognize and capture the oligosaccharides that will be transported into the cell through the SusC-like TonB-dependent transporter. A hallmark of canonical PULs is the presence of at least one sequential pair of SusC and SusD homologs (23). These proteins were shown to form complexes responsible for substrate uptake through a “pedal bin” mechanism (24–26). The SusC-like transporter acts as the barrel of the bin, whereas the SusD-like protein sits atop the barrel, opening and closing like a lid to facilitate substrate binding. SusD-like proteins are of utmost importance, as their deletion, whether or not it binds the cognate substrate, leads to a loss of growth (27–29).

The majority of the SusD-like proteins characterized so far come from cultivable *Bacteroides*, i.e. *B. thetaiotaomicron* (27, 30–33), *B. ovatus* (34–36), *B. fluxus* (37) or *B. uniformis* (37). To our knowledge, only one study characterized two SusD-like proteins that have been identified from an uncultured cow rumen bacterium (38). With regard to the diversity and importance of uncultured bacteria in the rumen, further studies are required to better understand the role of polysaccharide utilization loci in ruminal fiber degradation.

In a recent publication (39), activity-based metagenomics was used to identify the key enzymes involved in the breakdown of lignocellulosic substrates by two bacterial consortia derived from the bovine rumen. In this study, we further investigate the functional abilities of the clone 41O1 identified from the cow rumen microbiome after *in vitro* enrichment (IVTE) on wheat straw.

## RESULTS

### Functional annotation of the 41O1 metagenomic DNA sequence reveals a PUL likely involved in β-glucan utilization

**Fig 1:**
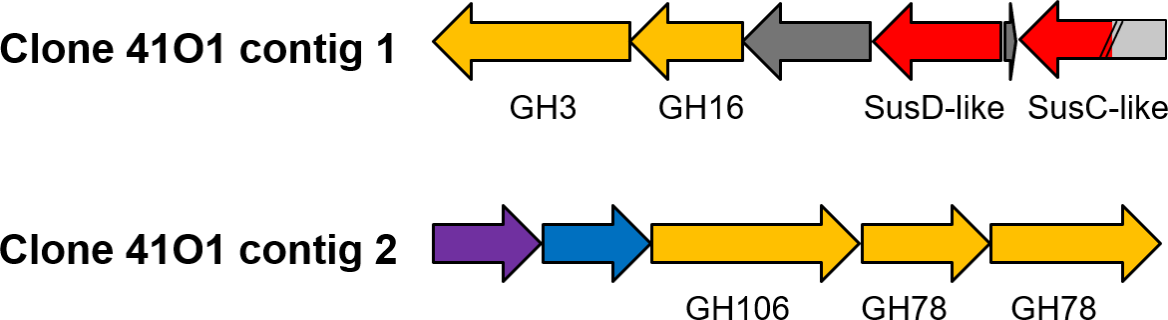
Representation of the 41O1 contigs. Genes encoding known and predicted functionalities are color-coded: glycoside hydrolase (GH) with family number in yellow; *SusD-like* and *SusC-like* genes in red, and genes with unknown function in dark gray; l-rhamnose-proton symporter in purple; rhamnulose-1-phosphate aldolase in blue. The *SusC-like* gene is artificially truncated by cloning of the metagenomic DNA fragment in the pEPIFOS-5 fosmid. The missing part is highlighted in light gray.

In the study by Ufarté *et al.* (39)*,* sequencing of the 41O1 clone using the MiSeq technology revealed two contigs that could not be assembled, as was also the case for 12 of the 26 sequenced clones in the metagenomic library. In this study, we thus performed long-read sequencing of 41O1 using the MinION Oxford Nanopore Technologies to get the full-length, circular sequence. Assembly results yielded a fosmid sequence of 47,880 bp with the presence of two inserts, yet separated by two full-length pEPIFOS-5 backbones (Fig. S1). These data were confirmed by enzymatic digestion of the fosmid (Fig. S2). Functional annotation of the contig_1 (22,594 bp) revealed a PUL containing genes encoding a glycoside hydrolase of family 3 (GH3), a member of glycoside hydrolase family 16 (GH16), a surface glycan-binding protein (SusD-like protein), and a TonB-dependent transporter (SusC-like protein) that is artificially truncated by the cloning (noted at one end of the DNA insert) (Fig. 1). The contig_2 (10,243 pb) contains genes encoding an l-rhamnose-proton symporter, a rhamnulose-1-phosphate aldolase, a member of the glycoside hydrolase family 106 (GH106) and two members of the glycoside hydrolase family 78 (GH78). The activities described in the CAZy database (http://www.cazy.org/) for both GH78 and GH106 are rhamnogalacturonan α-l-rhamnohydrolase and α-l-rhamnosidase.

### The 41O1_PUL confers the ability to grow on β-1,3/1,4- gluco-oligosaccharides to *E. coli*

**Fig 2:**
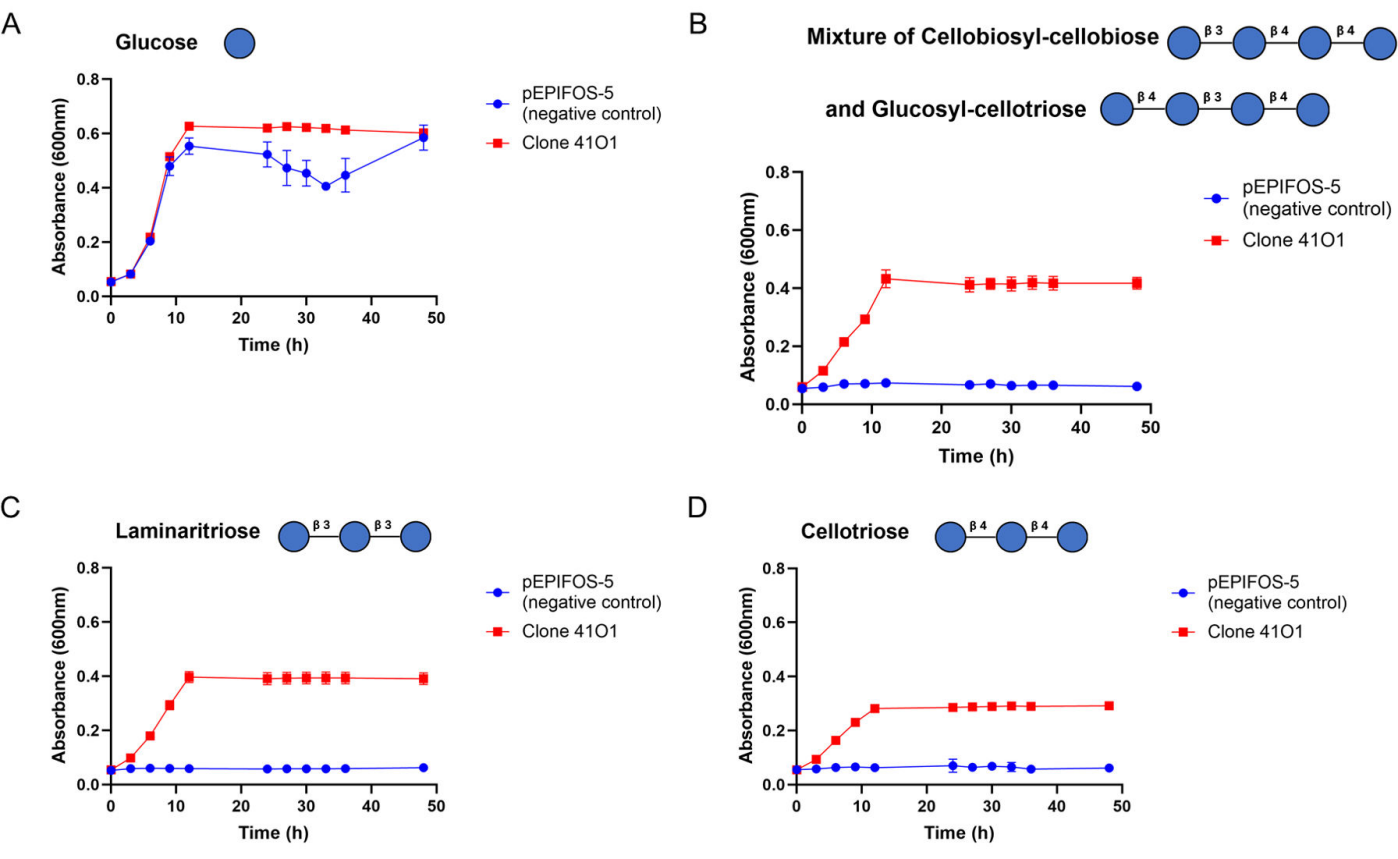
Growth curves of the clone 41O1 on β-1,3/β-1,4-linked gluco-oligosaccharides at 0.5% (w/v), highlighting significant growth ability on laminaritriose and a mixture of β-d-cellobiosyl-cellobiose and β-d-glucosyl-cellotriose, and slight growth is observed with cellotriose. pEPIFOS-5 (negative control) corresponds to *E. coli* transformed with the empty pEPIFOS-5 fosmid. The data represent the average of at least biological triplicates (three different single colonies).

According to the CAZy database, GH3 and GH16 are polyspecific families with more than 15 activities listed so far, including β-glucosidase, endo-β-1,3(4)-glucanase, xylanase and β-1,4-xylosidase activities. To screen the specificity of the 41O1_PUL CAZymes and SusD-like protein towards oligosaccharides of various structures, we performed growth assays in minimal medium containing, as the sole carbon sources, either β-gluco-oligosaccharides or xylo-oligosaccharides. Growth curves (Fig. 2) show that the 41O1 metagenomic clone exhibits significant growth ability when supplied with laminaritriose (β-1,3 linkages only) and a mixture of β-d-cellobiosyl-cellobiose and β-d-glucosyl-cellotriose (linear gluco-oligosaccharides containing both β-1,4 and β-1,3 linkages). Slight growth was observed with cellotriose (β-1,4 linkages only). No growth was observed with xylo-oligosaccharides (data not shown), highlighting the specificity of 41O1_PUL towards β-1,3 and β-1,4-linked gluco-oligosaccharides and its preference for β-1,3-linkages.

### Cellular localization of the clone activity

**Fig 3:**
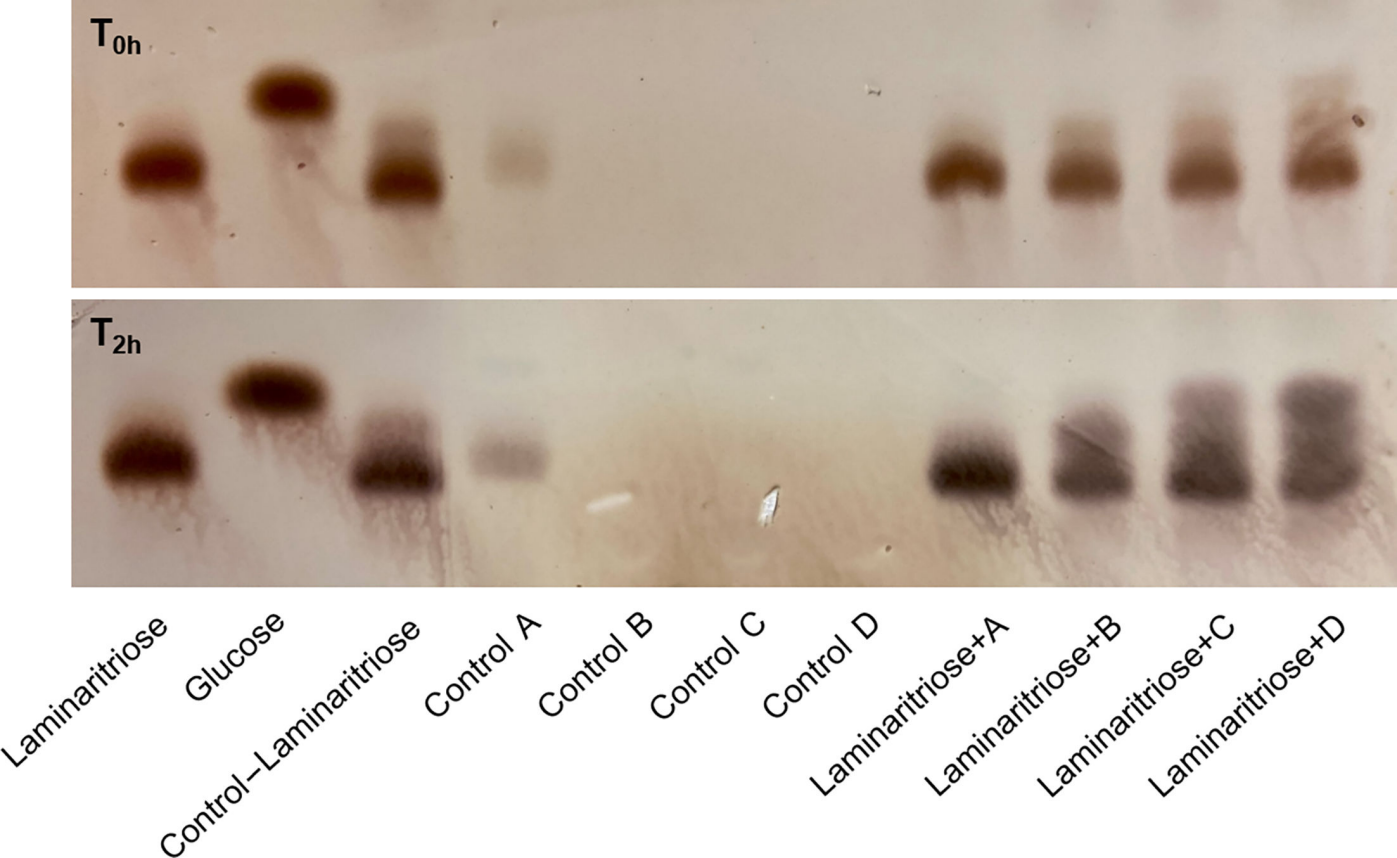
Cellular localization of enzymatic activity based on analysis of oligosaccharide hydrolysis reaction products by TLC, suggesting that clone 41O1 presents both membrane-bound and cytoplasmic β-1,3-glucosidase activities. Laminaritriose (β-1,3) hydrolysis reaction products were analyzed in four fractions: A – secreted fraction; B – whole cells; C – soluble intracellular fraction; D – membrane fraction. The control line “control–laminaritriose” corresponds to the substrate solution (0.2% (w/v) final concentration) and 50 mM potassium phosphate buffer, incubated overnight (about 20 h). Controls A–D correspond to the different cellular fractions, without any substrate, incubated overnight (about 20 h).

The growth phenotype indicates that the clone 41O1 produces at least one enzyme able to degrade gluco-oligosaccharides containing β-1,3 and β-1,4 linkages. Using SignalP 6.0 (40), we investigated the putative subcellular localization of the GH3 and GH16 enzymes encoded by the 41O1_PUL. Notably, a lipoprotein signal peptide (Sec/SPII) located at the N-terminal was predicted for both proteins, indicating their potential attachment to the bacterial membrane. In the native bacterium, these proteins are likely anchored to the outer membrane and surface-exposed, as several proteins from the Sus system have been shown to face the outside environment (41–43). To experimentally investigate the subcellular localization of the GH3 and GH16 enzymes produced by the clone 41O1, enzymatic assays on laminaritriose were performed using the secreted, whole cells, soluble intracellular and membrane protein fractions. The hydrolysis reactions involving these oligosaccharides were subjected to an incubation period of 2 h, after which the resultant products were resolved on thin-layer chromatography (TLC) plates. The outcomes obtained from TLC analysis most notably indicated significant breakdown of laminaritriose (β-1,3) to laminaribiose and glucose by both the soluble intracellular and the membrane fractions (Fig. 3). After 2 h, significant amounts of laminaribiose also appeared with whole cells, but there was no clear presence of glucose, probably because it has been metabolized by the living cells. Although the experimental setup does not preclude cross-contaminations between the different cellular fractions, the overall data suggest that clone 41O1 presents both membrane-bound and cytoplasmic β-1,3-glucosidase activities.

### The 41O1_PUL CAZymes prefer β-1,3-glucosyl linkages over β-1,4 and β-1,6 linkages

**Fig 4:**
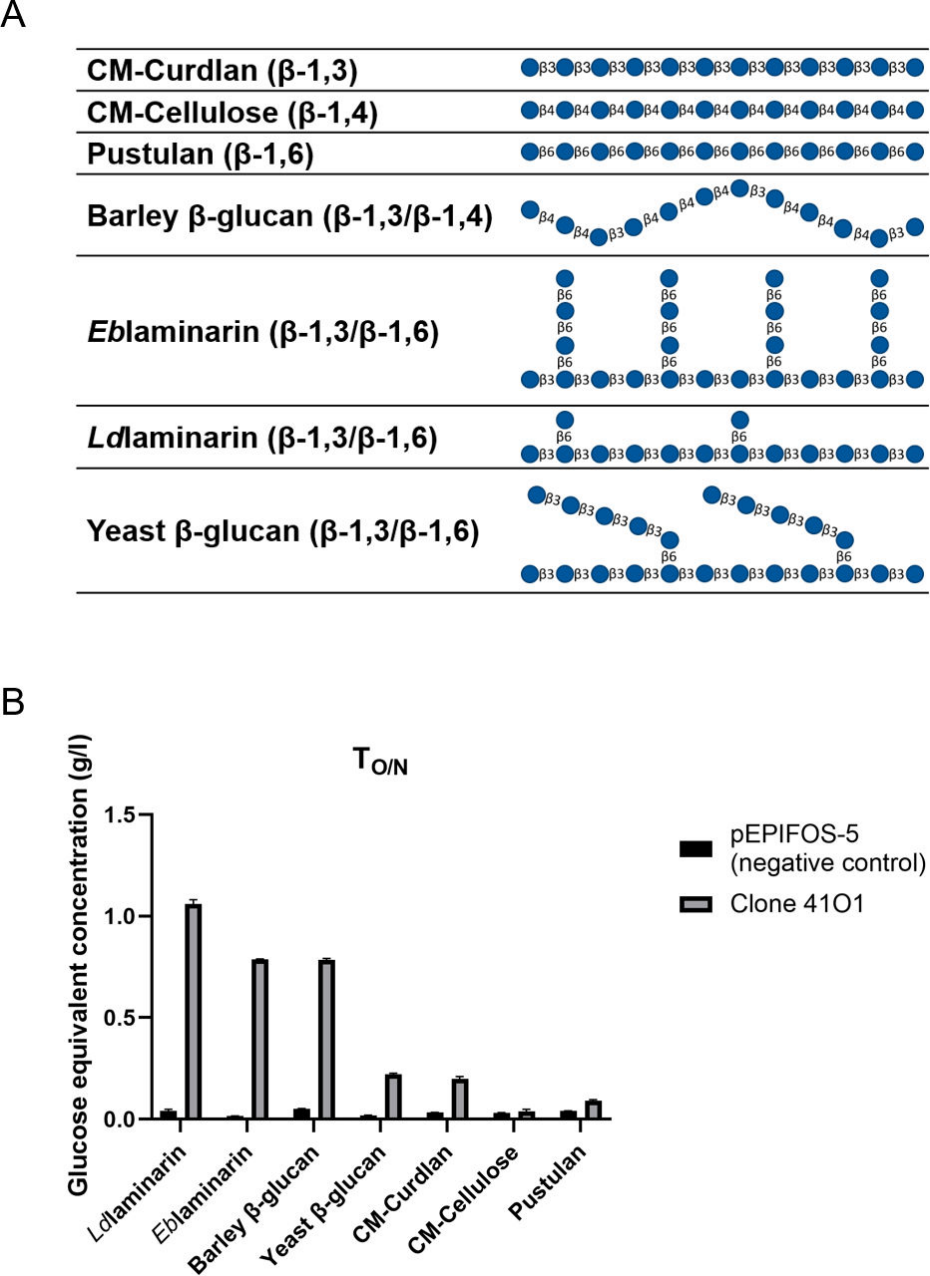
Enzymatic activities of clone 41O1, highlighting significant activity of the GH3 and/or the GH16 proteins on laminarins and barley β-glucan. (**A**) Structure of the different β-glucans used in this study. (**B**) Enzymatic activities of the soluble intracellular extract on various 0.2% (w/v) β-glucans were determined by DNS assays. *n* = 3, error bars represent the standard deviation of the means.

To investigate the preference of the 41O1_PUL CAZymes for β-glucans, we evaluated the hydrolytic activity of an intracellular extract of the metagenomic clone on polysaccharides using the 3,5-dinitrosalicylic acid reducing sugar (DNS) assay. The structure of the different polysaccharides is shown in Fig. 4A. As shown in Fig. 4B, significant hydrolytic activity was observed with the laminarins [*Ld*laminarin, from *Laminaria digitata*, *Eb*laminarin, from *Eisenia bicyclis*, linear β-1,3-linked glucans with some 6-O-branching in the main chain and some β-(1,6) intrachain links are also present (44)], and barley β-glucan (a linear chain of β-1,4-linked cellotriosyl and cellotetraosyl units linked by β-1,3 bonds) (45). Albeit to a lesser extent, enzymatic activity is also detected with yeast β-glucan [β-1,3-glucan containing β-1,3-glucan branches extending from β-1,6-linked branch points (46)] and CM-Curdlan (linear β-1,3). Conversely, no significant activity was observed with CM-Cellulose (β-1,4) and pustulan (β-1,6). This experiment supports the hypothesis that the GH3 and/or the GH16 proteins from the 41O1_PUL are involved in the growth phenotype on β-1,3/1,4-linked gluco-oligosaccharides.

### 41O1_SusD-like protein has greater binding affinity for β-1,6 branched β-1,3-glucans

Because the growth phenotypes and enzymatic activity profile of clone 41O1 are likely due to the 41O1_PUL, we wanted to investigate the substrate specificity of the associated SusC/D transporter. With the *41O1_SusC-like* gene being truncated, we focused characterization on the 41O1_SusD-like protein (protein sequence available in Fig. S3A). Indeed, prior investigations have provided compelling evidence highlighting the integral role of SusD-like proteins within the SusC/D transport system, particularly in the context of substrate uptake (28, 35, 47). The recombinant protein of 57 kDa was produced in soluble form in *E. coli* BL21(*DE3*) strain. The protein was purified to electrophoretic homogeneity by nickel affinity chromatography. The size exclusion chromatography analysis suggested that 41O1_SusD-like forms monomers and dimers in solution (Fig. S3B). We then investigated the binding abilities of the monomers towards different β-glucans.

**Fig 5:**
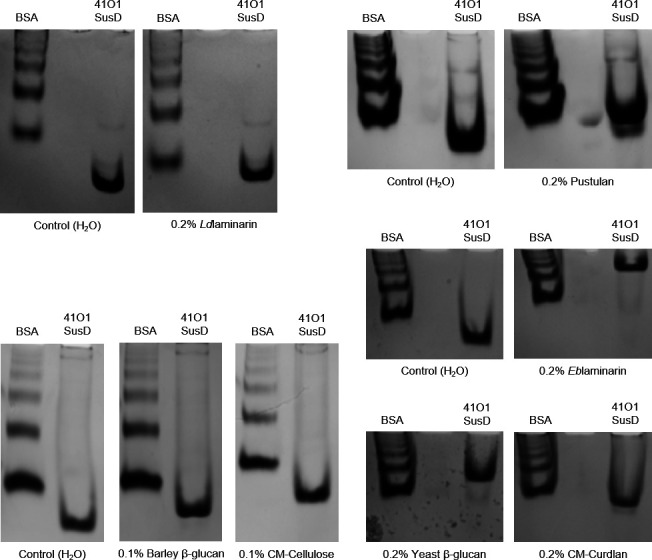
Affinity gel electrophoresis of 41O1_SusD-like on a gel containing no polysaccharide (control with H_2_O) and on gels with different β-glucans, with bovine serum albumin (BSA) as a control protein. The gel profiles suggest that 41O1_SusD-like has a greater affinity for β-1,6-branched β-1,3-glucans.

We first tested the binding ability of the 41O1_SusD-like protein towards soluble polysaccharides at 0.2% (w/v) (otherwise stated) using affinity gel electrophoresis (AGE). As depicted in Fig. 5, the migration of the 41O1_SusD-like protein was notably impeded in the presence of different β-1,3-glucans containing occasional β-1,6-linked glucose branches. Specifically, the gels containing *Ld*laminarin, *Eb*laminarin, and yeast β-glucan exhibited percentages of ~27%, 68%, and 45% retention of the 41O1_SusD-like protein, respectively. The molecular weights of the *Ld*laminarin and the *Eb*laminarin are in the same range (~6 kDa) (48), so that this cannot explain the shift difference on the gels. Nevertheless, the structure of these polysaccharides can vary according to the season, and a study identified an *Eb*laminarin of high molecular weight (up to 27 kDa) when *E. bicyclis* is harvested in July (49). The season in which the substrate is prepared by the supplier could affect the migration profile. It is worth noting that *Eb*laminarin contains more β-1,6-linked side chains compared with *Ld*laminarin, and these branches can display a degree of polymerization up to three glucose units linked in β-1,6 (49, 50). To test whether 41O1_SusD-like could target β-1,6 linkages, we performed AGE using pustulan, a linear β-1,6-glucan. The results in Fig. 5 show a retention of ~12%, indicating that it is unlikely to be the primary substrate motif targeted by 41O1_SusD-like. As mentioned earlier, the yeast β-1,3-glucan contains β-1,3-glucan branches extended from β-1,6-linked branch points (46). 41O1_SusD-like displays a significant shift on this polysaccharide (~ 45%), whereas it is much less marked (~20%) on the high-molecular-weight linear β-1,3-glucan CM-Curdlan (1,650 kDa; Megazyme). We cannot exclude the fact that the weaker shift on CM-Curdlan is due to the presence of the carboxymethyl groups that enable the substrate to be soluble. Because these substitutions might interfere with the protein–carbohydrate binding interactions, we could not conclude on the 41O1-SusD-like ability to bind unmodified curdlan using this experimental setup. In addition, there is no significant retention on barley (1,3;1,4)-β-glucan (~6%) and CM-Cellulose (~0%) at 0.1% (w/v). These data suggest that 41O1_SusD-like has a greater affinity for β-1,6-branched β-1,3-glucans.

We also assessed the binding ability of 41O1_SusD-like towards soluble polysaccharides by thermal shift analysis (TSA). In this experiment, we monitored the impact of the various ligands on temperature-induced protein unfolding. An increased thermal stability in the presence of a ligand was detected by a discernible shift of the unfolding profile (brightness ratio) towards a higher inflection temperature (Ti) compared with the profile of the protein without ligand. Our results are presented in Table 1; Fig. S4. Specifically, the Ti of 41O1_SusD-like is higher in the presence of *Ld*laminarin, *Eb*laminarin, and yeast β-glucan with increments of +2.6°C, +8.9°C, and +6.5°C, respectively. Minor impacts on the thermal stability can be observed in presence of CM-Curdlan (+1.2°C) and pustulan (+1.6°C), whereas almost no significant changes are recorded in the presence of barley β-glucan (+0.7°C) and CM-Cellulose (+0.4°C). These results are in line with the AGE experiments and corroborate the hypothesis that 41O1_SusD-like targets β-1,6-branched β-1,3-glucans.

**TABLE 1:** Thermal shift analysis of 10 µM 41O1_SusD-like protein incubated with 0.5% (w/v) ligands

Substrates	∆T°C
Laminaritriose	4.7
Cellotriose	1.2
Mixture of cellobiosyl-cellobiose and glucosyl-cellotriose	2.6
*Ld*laminarin	2.6
*Eb*laminarin	8.9
Yeast-β-glucan	6.5
Barley-β-glucan	0.7
CM-Curdlan	1.2
CM-Cellulose	0.4
Pustulan	1.6

TSA was also used to investigate the binding ability of the protein towards the β-gluco-oligosaccharides that were used in the growth assays. Laminaritriose and the mixture of glucosyl-cellotriose and cellobiosyl-cellobiose display the highest shift in the Ti, with an increment of +4.7°C and +2.6°C, respectively. The presence of cellotriose only produces a minor impact on the Ti, with an increment of +1.2°C. These data suggest that 41O1_SusD-like has a slight ability to interact with β-1,4-linked oligosaccharides but shows a stronger affinity for the oligosaccharides containing β-1,3-linkages. Several studies on the canonical SusD protein (27) and other SusD-like proteins (36, 37) have shown that the affinity increases with degree of polymerization of the cognate oligosaccharides. Therefore, additional experiments using oligosaccharides with degrees of polymerization higher than four would enable to further investigate the binding preference of 41O1_SusD-like.

**Fig 6:**
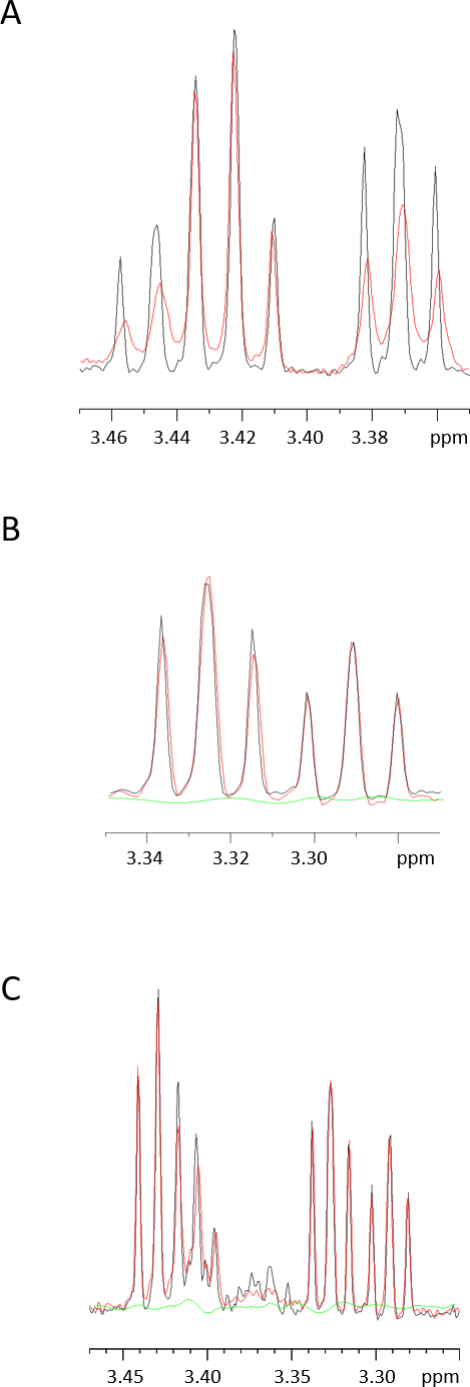
Characterization of the ligand-binding abilities of 41O1_SusD-like using NMR spectroscopy, demonstrating the binding affinity of 41O1_SusD-like for laminaritriose. (**A**) 1D NMR spectrum of the laminaritriose centered on the signals at 3.46–3.36 ppm: Black – Reference 1D proton spectrum of 1 mM laminaritriose; Red - 1D proton spectrum of the same laminaritriose in the presence of 10 µM 41O1_SusD-like. (**B**) Same as panel A but for the cellotriose, proton spectrum centered on the signals at 3.34–3.28 ppm. The green spectrum is the STD signal of the same cellotriose with and without protein saturation. (**C**) Same as panel A but for the mixture of glucosyl-cellotriose and cellobiosyl-cellobiose, proton spectrum centered on the signals at 3.46–3.26 ppm. The green spectrum is the STD difference spectrum of the mixture with and without protein saturation.

Finally, we corroborated these results using NMR spectroscopy. The 1D NMR spectra of the different putative ligands in the absence (black) and presence (red) of 41O1_SusD-like are shown in Fig. 6. The 1D proton spectrum corresponding to laminaritriose exhibits a pronounced line, broadening in the presence of 41O1_SusD-like (Fig. 6A). This is indicative not only of a discernible molecular interaction between both molecules but also of the time scale of this interaction. Indeed, to obtain line broadening despite the 100:1 excess of ligand, exchange between bound and free form cannot be too rapid. Saturation transfer difference (STD) NMR spectra (51) of the same samples were not successful, probably due to the slow exchange. Extending the analysis to the other putative ligands, we observed no obvious influence on the 1D proton peaks of cellotriose in the presence of 41O1_SusD-like, and the STD spectrum was equally flat (Fig. 6B). In the case of the mixture of cellobiosyl-cellobiose and glucosyl-cellotriose (Fig. 6C), the major peaks were not influenced by the presence of the protein, although some minor components with signals between 3.35 and 3.40 ppm did show selective broadening.

In summary, our investigations applying different methodologies have demonstrated the clear binding affinity of 41O1_SusD-like for β-1,6 branched β-1,3-glucans and laminaritriose.

### 3D structure analysis of 41O1_SusD-like

**Fig 7:**
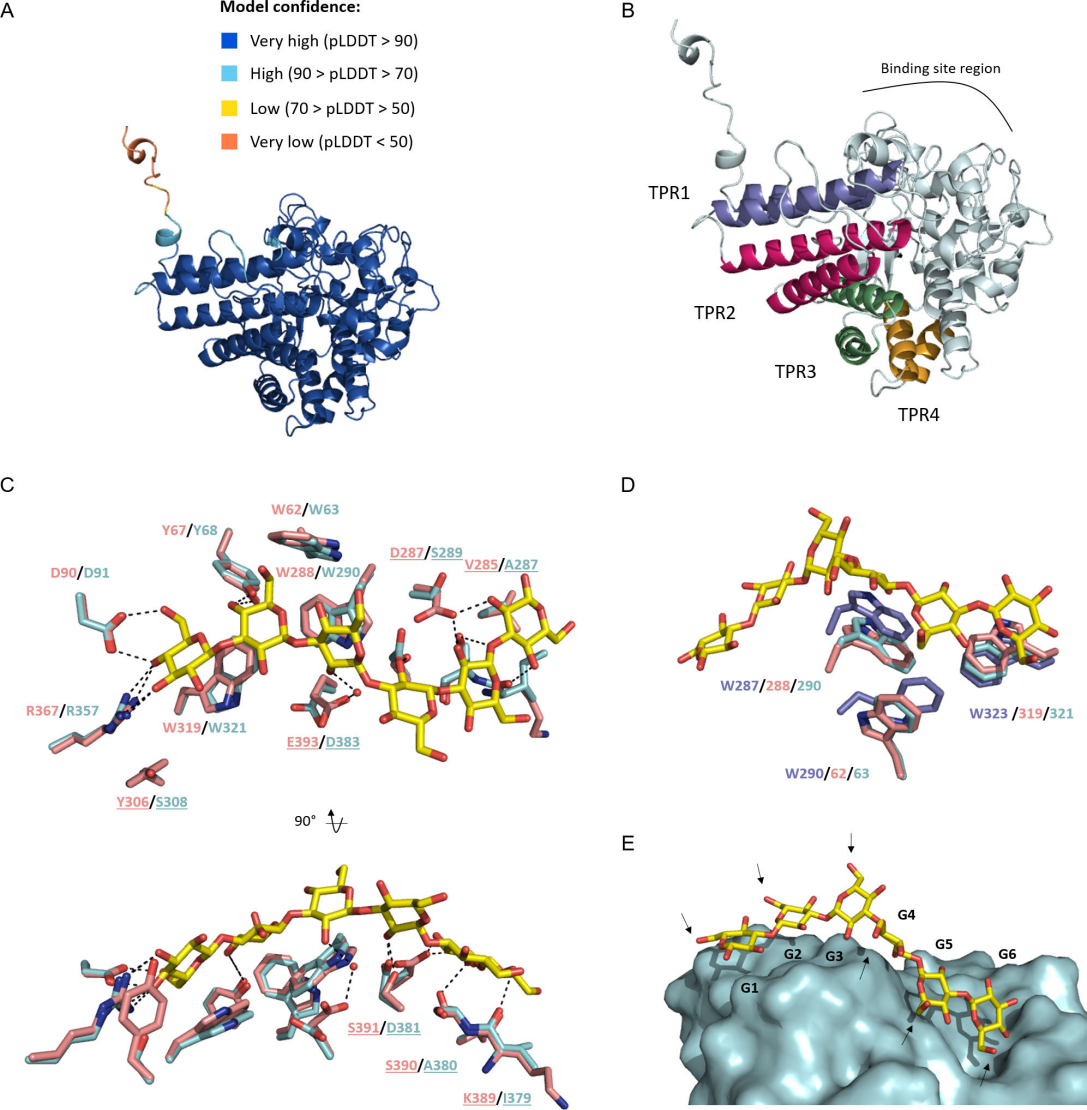
Structural analysis of the best AF2 model of 41O1_SusD-like mature protein. (**A**) Quality assessment of the best AF2 model of 41O1_SusD-like. The protein is shown in cartoon model and colored according to pLDDT confidence score. (**B**) 41O1_SusD-like displays the canonical “RagB/SusD” fold. TPR (tetratricopeptide repeats) domain analysis. TPR domains are highlighted in colors and labeled onto the overall 3D structure in cartoon model. (**C**) Structural comparison of the binding site. 41O1_SusD-like (palecyan sticks) was superimposed onto *Bt*SGBP-A (light pink sticks) in complex with laminarihexaose (yellow sticks) (PDB ID: 7KV3). The residue names are labeled and colored according to the sequence. The residues that differ between the two structures are underlined. Two views rotated at 90° are displayed. Hydrogen bonds between residues and sugars are shown in dashed black lines. (**D**) Zoomed view on the three key tryptophans binding the laminarihexaose (yellow sticks). Three 3D structures were superimposed with 41O1_SusD-like in palecyan sticks, with *Bt*SGBP-A in light pink sticks and GM_SusD in slate sticks (PDB ID: 6GCZ). (**E**) Superimposition of the laminarihexaose from 7KV3 on the surface of 41O1_SusD-like. Black arrows point to the C6-OH of each monosaccharide and represent space where β-1,6-linked branches can be accommodated. The residue numbering corresponds to the full-lenght protein sequences found in the UniProt database (*Bt*SGBP-A: A0A173VPY3, GM_SusD: A0A1H1MMX0).

To investigate the molecular determinants of substrate recognition by 41O1_SusD-like, we used crystallogenesis in an attempt to determine its structure by X-ray crystallography. Unfortunately, we were unsuccessful in obtaining crystals, so its 3D structure was modeled using the AlphaFold2 (AF2) algorithm from LocalColabFold (52, 53). All the AF2 models showed high confidence scores with pLDDT values ranging from 95.4 to 95.8 for the mature protein, on average. All the statistics and metrics for the full length AF2 models, such as pLDDT, PAE and coverage, are given following this link https://doi.org/10.5281/zenodo.10931522. The 3D structure of the best AF2 model of 41O1_SusD-like mature protein is shown in Fig. 7A. To go further in the understanding of the 3D model, a BLASTp search was performed against the Protein Data Bank (PDB) (https://blast.ncbi.nlm.nih.gov/Blast.cgi?PAGE=Proteins). The closest homolog was found to be a protein of RagB/SusD family from *B. thetaiotaomicron* (*Bt*SGBP-A) (PDB ID: 7KV2) (37). Interestingly, the template sequence (7KV2) covered 100% of 41O1_SusD-like length, with an e-value of 0.00 and 70.99% of sequence identity. As expected, this sequence was used as one template among others by AF2. To compare both 41O1_SusD-like and 7KV2 structurally, the two 3D structures were aligned using the TM-align server (https://zhanggroup.org/TM-align/) (54). The RMSD and TM-score values were of 0.47 Å and 0.9, respectively, indicating that 41O1_SusD-like has the same fold as 7KV2, which corresponds to the canonical “RagB/SusD” fold. This latter is composed of one SusD-like domain (PFAM PF14322) and one SusD-RagB domain (PFAM PF07980). Based on this information, the conserved tetratricopeptide repeat (TPR) units (24, 27) known for this family were determined by comparing 41O1_SusD-like with the X-ray structure of SusD from *B. thetaiotaomicron* (PDB ID: 3CKC) (27); this is shown in Fig. 7B. Eight α-helices were found to pack together and form a right-handed superhelix along one side of the structure. These were clustered in TPR domains, such as TPR1: α1 (residues 44–60) and α4 (residues 110–133); TPR2: α5 (residues 139–164) and α6 (residues 185–202); TPR3: α7 (residues 219–233) and α8 (residues 236–252); and TPR4: α16 (residues 395–408) and α17 (residues 415–426).

To investigate the binding site region of 41O1_SusD-like, X-ray structures of SusD-like proteins were searched in complex with carbohydrates. Interestingly, the structure of *Bt*SGBP-A was solved in a bound-form with laminarihexaose (PDB ID: 7KV3), pointing to binding at the non-reducing end in an exo-mode (37). Moreover, *Bt*SGBP-A has been shown to bind to *Ld*laminarin, *Eb*laminarin and yeast β-glucan (55). Therefore, by superposing the 41O1_SusD-like model and the *Bt*SGBP-A X-ray structure (PDB ID: 7KV3), it can be seen that the key residues involved in hydrogen bonds at the non-reducing side of the oligosaccharide are conserved (Y68, D91 and R357 for 41O1_SusD-like) (Fig. 7C). More differences occur at the reducing side. S390 (*Bt*SGBP-A) is replaced by an alanine in 41O1_SusD-like (A380), leading to a loss of hydrogen bond by the serine hydroxyl group. Interestingly, a swap occurs in which D287 (*Bt*SGBP-A) is replaced by S289 (41O1_SusD-like), and S391 (*Bt*SGBP-A) is replaced by D381 (41O1_SusD-like). Finally, the glutamate E393 in *Bt*SGBP-A is replaced by an aspartate D383 (41O1_SusD-like), both of which could form an indirect hydrogen bond with the ligand via a water molecule. With regard to hydrophobic interactions, three tryptophans (W63, W290, and W321 for 41O1_SusD-like) seem to be key to binding (Fig. 7D). These are conserved in both sequence and structure when compared with *Bt*SGBP-A (W62/W288/W319) (Fig. 7D). To take things further, we compared 41O1_SusD-like with a SusD from the marine Bacteroidota member *Gramella* sp. MAR_2010_102 (GMSusD) (PDB ID: 6GCZ), which is known to bind to branched laminarin (50). Both sequences share 29% sequence identity. Surprisingly, only two of three tryptophans aligned in terms of primary sequence and 3D structures, namely W290/W287 and W321/W323 for 41O1_SusD-like and GMSusD respectively. The third one, W290 for GMSusD, is close spatially to W63 for 41O1_SusD-like, but is not located in the same region in the primary sequence. Interestingly, site-directed mutagenesis of the tryptophan side chains from GMSusD (W287, W290 and W323) plays a critical role in substrate recognition (50), indicating the importance of this triad at the binding site. Finally, superimposing 41O1_SusD-like and *Bt*SGBP-A in complex with laminarihexaose revealed a free space around the C6-OH group at each glucose binding site (Fig. 7E), thereby providing an explanation for its substrate versatility, especially towards β-1,6-branched β-1,3-glucans.

## DISCUSSION

In this study, we have investigated the functional abilities of metagenomic 41O1_PUL that confers on *E. coli* the ability to grow on β-1,3/1,4-gluco-oligosaccharides. The functional annotation revealed that 41O1_SusC-like is 40% truncated on the N-terminal side when compared with SusC homologs, ruling out the 41O1_SusC-like transporter being involved in the substrate uptake. Nevertheless, the investigation of the cellular localization of the enzymatic activity revealed the presence of at least one enzyme at the cell surface. We showed that laminaritriose is broken down into laminaribiose by the whole cells, which likely metabolize the released glucose. The presence of enzymatic activities at the cell surface therefore explains the ability of the clone 41O1 to grow on the oligosaccharides, whereas its SusC-like transporter is truncated. In *E. coli*, it is widely believed that nearly all lipoproteins are either retained in the inner membrane or transferred to the inner leaflet of the outer membrane (56). Nevertheless, the free-form of the lipoprotein Lpp, the most numerically abundant protein in *E. coli*, has been shown to be surface-exposed (57) and, generally speaking, the prevalence of the surface-exposed lipoproteins in Gram-negative bacteria has been underestimated (58).

The detection of enzymatic activities in the soluble intracellular fraction (containing both the cytoplasm and the periplasm) is rather intriguing as both GH3 and GH16 proteins display a predicted lipoprotein signal peptide. In our context, it is possible that these native peptide signals are not well recognized by *E. coli,* so translocation to the membrane does not occur.

In a previous study (28), we showed that the genes are spuriously transcribed in *E. coli* due to the random presence of *E. coli* rpoD/r70 promoter sequences on metagenomic DNA inserts. In this paper, our experiments do not allow us to know whether the *41O1-SusD-like* gene is transcribed and the corresponding protein located at the cell surface. Therefore, further experiments are required to evaluate its expression and its possible role in tethering of glycans to the cell surface.

Superimposing the 3D structures of 41O1_SusD-like, *Bt*SGBP-A and GMSusD highlights a similar pattern of β-1,3-glucans recognition, although they come from different environmental niches. Examples of gene transfer between such different environments have previously been described with the transfer of carbohydrate utilization systems from marine to human gut bacteria (59–61). Nevertheless, 41O1_SusD-like (from the cow rumen) and *Bt*SGBP-A (from the human gut) share a high percentage of sequence identity (71% ID), although they come from environments that are not in contact with one another. Furthermore, a structural alignment showed that the three tryptophans are located at similar sequence positions within 41O1_SusD-like (**W63**, W290, and W321) and *Bt*SGBP-A (**W62**, W288, and W319), whereas in GMSusD (from the marine environment) (**W290**, W287, and W323), one of the tryptophans is located at a completely different sequence position. This is made possible due to a ‘swap’ between residues in contact (i.e. that are close spatially), to offset the changes. In this instance, the residue pair **W63**/P293 in 41O1_SusD-like is replaced by G63/**W290** in GMSusD. Overall, these results suggest that this substrate binding site topology, most notably an aromatic triad, is efficient for β-1,3-glucans recognition and might have appeared several times during evolution.

Deciphering which substrate is targeted by a PUL is always a laborious task that involves the use of several approaches. Although there are no metatranscriptomic data available that could guide us to identify in which substrates conditions our PUL is upregulated, we can still rely on biochemical data to draw hypotheses on the substrate targeted by 41O1_PUL. The 41O1 CAZymes and SusD-like protein exhibit clear enzymatic activity and binding respectively, towards the *Eb*laminarin (1,3;1,6)-β-d-glucan. In addition, the AlphaFold2 model of 41O1_SusD-like provides an explanation for its affinity towards *Eb*laminarin, with the C6-OH groups of the glucosyl residues oriented to the solvent. However, in the context of the cow rumen, this polysaccharide from brown algae is unlikely the targeted substrate. A more probable substrate is a (1,3;1,4)-β-d-glucan that is present in cereals grains (62), which are part of the cow diet (63). A clear enzymatic activity is detectable using barley β-glucan, but we showed that 41O1_SusD does not bind to this polysaccharide and only slightly to the corresponding oligosaccharides. Nevertheless, this does not mean that it is not the substrate taken up by the 41O1_SusC/D transporter, as exemplified by the F5_SusD from a xylo-oligosaccharide (XOS) targeting PUL: this has been shown not to be able to bind to XOS or xylan (29). Indeed, regarding the recent structural insights showing that SusC-like and SusD-like proteins are closely associated *in vivo* (24, 26, 64), it is reasonable to hypothesize that SusD-like proteins that cannot bind glycans as isolated proteins can actually do it in the presence of the cognate SusC-like protein. Another hypothesis would be that the 41O1_PUL actually targets the (1,3;1,6)-β-d-glucans from the cell wall of the yeasts naturally present in the rumen, such as *Candida* species (65, 66), which are facultative anaerobes belonging to the Ascomycota phylum, or from dietary yeasts. In this study, the metagenomic DNA was indeed sampled from the rumen of cows of which the diet contained corn silage (39), which itself contains *Saccharomycetales* (67). The targeted β-glucans might also come from the strict anaerobic fungi that are part of the rumen microbiota. Presently, these anaerobic fungi all belong to the early-branching phylum Neocallimastigomycota (68), and they are attracting increasing attention for their functional contribution to the digestion of recalcitrant plant fiber (12). Nevertheless, to our knowledge, no study has been conducted on the presence of β-glucan in their cell wall and the only study we were able to find concerned the identification of chitin (69).

Determining the substrate targeted by the 41O1_PUL will require fine biochemical characterization of the GH3 and GH16 proteins on well-defined substrates. In addition, just downstream of 41O1_SusD-like, there is a protein of unknown function. Its sequence analysis reveals the presence of a lipoprotein signal peptide, suggesting that the mature protein is found anchored to the outer membrane and facing the environment. Its gene locus inside the PUL, as well as the presence of a lipoprotein signal peptide, indicate that this protein is likely an SGBP-B, with 41O1_SusD-like being the SGBP-A. Although the majority of the GHs and related SGBPs were shown to display concordant specificities (27, 32, 34–36, 70–72), recent studies have highlighted that the different specificities within GHs and SGPBs collectively dictate the overall specificity of the PUL (37, 55). Further investigation of the 41O1_PUL will therefore be needed, but our characterization of the 41O1_SusD-like paves the way for a better understanding of the carbohydrate metabolism by an uncultured bacterium from the cow rumen.

## MATERIALS AND METHODS

### Substrates

Barley β-glucan, carboxymethyl cellulose (4M), CM-Curdlan, and Yeast β-glucan were purchased from Megazyme (Bray, Ireland). Laminarin from *Laminaria digitata* (*Ld*laminarin) and laminarin from *Eisenia bicyclis* (*Eb*laminarin) were purchased from Sigma-Aldrich (St. Louis, MO, USA) and Biosynth Carbosynth (Berkshire, UK), respectively. Pustulan polysaccharide was purchased from Elicityl (Grenoble, France). Laminaritriose (G3G3G), mixture of 3^2^-β-d-Cellobiosyl-cellobiose (G3G4G) and 3^3^-β-d-Glucosyl-cellotriose (G4G3G), and cellotriose (G4G4G) were purchased from Megazyme (Bray, Ireland).

### Sequence of the metagenomic clone 41O1

The metagenomic clone 41O1 was issued from an *in vitro* enriched cow rumen metagenomic library (20,352 clones, *E. coli* EPI100, pEPIFOS-5 fosmid) (Ufarté *et al*., doi.org/10.1101/2024.03.15.585145). The long-read sequencing using the MinION Oxford Nanopore Technologies was performed by the GenoToul GeT-BioPUCE platform in Toulouse. The nucleotide sequence of the clone 41O1 is available in the GenBank database at http://www.ebi.ac.uk/ena/data/view/OZ022632-OZ022684, under accession number OZ022674.1. The 41O1_SusD sequence is available under accession number CAK9712120.1.

### Growth study

The clone 41O1 stored in glycerol at −80°C was recovered on Luria-Bertani (LB) agar plates supplemented with 12.5 mg/l chloramphenicol. Precultures of clone 41O1 were made from three isolated colonies added to liquid LB medium supplemented with 12.5 mg/l chloramphenicol and incubated overnight at 37°C with orbital shaking at 200 rpm. These precultures were used to inoculate 500 µl of M9 medium containing 0.5% (w/v) glucose, laminaritriose, cellotrios,e or a mixture of cellosyl-cellobiose and glucosyl-cellotriose at an initial OD_600_ of 0.05 into a 48-well microplate. The M9 medium contained (Na_2_HPO_4_·12H_2_O, 17.4 g/l; KH_2_PO_4_, 3.03 g/l; NaCl, 0.51 g/l; NH_4_Cl, 2.04 g/l; MgSO_4_, 0.49 g/l; CaCl_2_, 4.38 mg/l; Na_2_EDTA·2H_2_O, 15 mg/l; ZnSO_4_·7H_2_O, 4.5 mg/l; CoCl_2_·6H_2_O, 0.3 mg/l; MnCl_2_·4H_2_O, 1 mg/l; H_3_BO_3_, 1 mg/l; Na_2_MoO_4_·2H_2_O, 0.4 mg/l; FeSO_4_·7H_2_O, 3 mg/l; CuSO_4_·5H_2_O, 0.3 mg/l; thiamine, 0.1 g/l; and leucine, 0.02 g/l). Cell growth was followed by measuring the OD_600_ over 48 h at 37°C and 200 rpm.

### Cellular localization of the clone activity

To investigate the cellular localization of the glycoside hydrolases (GHs) encoded by the contig_1 of the clone 41O1, laminaritriose hydrolysis assays were performed using the different cellular fractions, as well as the extracellular fraction. The clone 41O1 was grown in 20 mL of LB at 37°C until OD_600_ reached 0.7–0.8. The cells were collected by centrifugation at 4°C for 10 min at 5,000 rpm. The supernatant was filtered (0.22 µm), and this fraction was called “secreted fraction”. The pellet was gently resuspended in 2 mL of 50 mM potassium phosphate buffer (PBS) (pH 7.0). 1 ml of this suspension was kept and called “whole cells” for the activity test. The remaining 1 ml was sonicated to lyse the cells. The lysate was centrifuged at 4°C for 10 min at 10,000 rpm. The supernatant was called “soluble intracellular fraction”. The pellet was resuspended in 1 ml 50 mM of PBS and was called “membrane fraction”. The reaction media, 400 µl in total, were prepared in 50 mM PBS pH7.0 using 200 µl of the different fractions supplemented by laminaritriose at a final concentration of 0.2% (w/v). The mixtures were incubated for 2 h at 37°C and 200 rpm.

Thin layer chromatography (TLC) experiments were performed using the plates from Sigma (Product 53356, silica gel on TLC Al foil, 20 cm X 20 cm, no fluorescent indicator). Subsequently, 3 µl of each sample was loaded on the TLC plates and dried using a hairdryer. The plates were inserted in a TLC tank containing the migration buffer [butan-1-ol/acetic acid/H_2_O (2:1:1, v/v/v)]. When the solvent migration front was near the top of the plates (0.5 cm), they were dried and put into the revelation buffer (1 g/l orcinol, 75% (v/v) ethanol, and 3% (v/v) sulfuric acid) for 1 min and shaken gently. The sugars were revealed at high temperature (>60°C) using a hairdryer.

### Enzymatic assays

A preculture of clone 41O1 was made from an isolated colony added to liquid LB medium supplemented with 12.5 mg/L chloramphenicol. It was incubated overnight at 37°C with orbital shaking at 200 rpm. The OD_600_ was measured, and the cells were harvested by centrifugation at 4°C for 10 min at 10,000 g. The pellet was re-suspended in 50 mM potassium phosphate buffer (pH 7.0) to obtain a final OD_600_ of 80. The cells were broken by sonication (Fisher Scientific sonicator) with 5 cycles of 20 s, separated by 4 min on ice using the probe at 30% of the maximal power. The samples were centrifuged at 20,000 g for 10 min at 4°C, and the supernatant was filtered with a 0.22 µm filter (Minisart) to remove cell debris. The solution obtained was used to perform the activity tests.

To evaluate substrate specificity, the release of reducing sugar was measured using the 3,5-dinitrosalicylic acid reducing sugar (DNS) assay (DNS solution: 10 g/l DNS, 300 g/l potassium sodium tartrate, 16 g/l NaOH in deionized water) (73). The assays were carried out at 37°C, 200 rpm, in 50 mM potassium phosphate buffer pH 7.0, containing 0.2% (w/v) of substrate and 160 µl of cellular extract. The total volume of the reaction medium was 400 µl. Standard curves were made with glucose concentrations ranging from 0 to 2 g/l. 120 µl of reaction was taken at T_0h_ and T_overnight_ and added to an equal volume of DNS reagent. The samples were boiled for 5 min and centrifuged for 1 min at 20,000 g. Subsequently, 200 µl of the supernatants was transferred into the wells of a polystyrene microplate to read the OD at 540 nm in an Optima (TECAN) plate reader. The experiments were carried out in triplicates.

### Recombinant protein production and purification

The DNA sequence corresponding to the 41O1_SusD-like protein was optimized for *E. coli* and synthesized by GeneCust. The lipoprotein peptide signal predicted by LipoP 1.0 (74) was not included in the sequence. The gene was cloned between the Ndel and Xhol restriction sites in the pET-28a(+) expression vector (Novagen, Darmstadt, Germany) that enables the fusion of a 6-His tag at the N-terminal for affinity purification.

The pET-28a (+) plasmid containing the 41O1_SusD-like gene was transformed into *E. coli* BL21(*DE3*) for protein production. A single bacterial colony was inoculated in 5 ml of LB medium containing kanamycin (50 µg/ml) and incubated at 37°C, 200 rpm. The overnight preculture was used to inoculate 200 ml of LB containing 50 µg/ml of kanamycin at an initial OD_600_ of 0.05. The overexpression of 41O1_SusD-like protein was induced by adding isopropyl β-d-1-thiogalactopyranoside (IPTG) to a final concentration of 0.5 mM in the mid-exponential phase (OD_600_ ≈ 0.6), and the culture was further incubated for 4 h at 37°C, 200 rpm. The cells were harvested by centrifugation at 5,000 rpm for 10 min at 4°C. The pellet was resuspended in Talon Buffer (20 mM Tris-HCl, 300 mM NaCl, pH 7.4) and disrupted by sonication. Cells debris were cleared by centrifugation at 11,000 g for 30 min at 4°C. The supernatant was further filtered using a 0.22 µm filter (Minisart) and passed through a column of 2 ml of Talon metal affinity resin (Clontech, USA). A washing step was performed with buffer containing 10 mM imidazole, 20 mM Tris-HCl, 300 mM NaCl, pH 7.4. The 41O1_SusD-like protein was eluted with 250 mM imidazole, 20 mM Tris-HCl, 300 mM NaCl, pH 7.4. The imidazole was further eliminated using a PD-10 Desalting Column (Cytiva), and Tris buffer (20 mM Tris-HCl, 150 mM NaCl, pH 7.4). Protein purity was confirmed via SDS-PAGE and the concentration was determined by the absorbance at 280 nm using the Thermo Scientific Nanodrop 2000 with an extinction coefficient of 143,390 M^−1^cm^−1^. The protein was then further polished by gel filtration using an AKTA System (HiPrep 16/60 Sephacryl S-200 HR) (GE-Healthcare, Uppsala, Sweden) in 20 mM Tris-HCl, 150 mM NaCl, pH 7.4. The fractions containing the monomeric and dimeric 41O1_SusD-like protein were pooled respectively, concentrated and stored at 4°C. The monomeric form of 41O1_SusD-like was used for analysis.

### Crystallization assays

Crystallization assays were attempted with 41O1_SusD-like monomers concentrated at 9.63 mg/ml. Initial crystallization conditions were screened using a Mosquito robot (TTP Labtech) and the commercial screens Classic Suite, JCSG+ and PACT from Qiagen. We also attempted to co-crystallize 41O1_SusD-like with 10 mM of laminaritriose using the same conditions.

### Affinity gel electrophoresis

To investigate the ability of 41O1_SusD-like to bind polysaccharides, affinity gel electrophoresis (AGE) was performed. Continuous native polyacrylamide gels were prepared consisting of 8% (w/v) acrylamide in 25 mM Tris, 250 mM glycine buffer, pH 8.3. Various polysaccharides were added before polymerization at a final concentration of 0.5% (w/v). Subsequently, 2.5 µg of the tested 41O1_SusD-like, along with bovine serum albumin (BSA) used as non-interacting negative control protein, was loaded on the gels. Electrophoresis was carried out at 90V on ice for at least 2 hours. The percentage of retention of 41O1_SusD-like in the gel containing glycans was calculated as described by Tauzin et al. (29): (D_wog_ - D_wg_)/(D_wog_) × 100, where D_wog_ = D_SusD-like_wog_/D_BSA_wog_ (distance of migration of the lowest and most intense 41O1_SusD-like band normalized to the distance of migration of BSA in the gel without glycan), and D_wg_ = D_SusD-like_wg_/D_BSA_wg_ (distance of migration of the lowest and most intense 41O1_SusD-like band normalized to the distance of migration of BSA in the gel with glycan).

### Thermal shift analysis

Thermal shift analysis (TSA) is a rapid and inexpensive label-free screening method to identify low-molecular-weight ligands that bind and stabilize purified proteins. The structural stability of 41O1_SusD-like in the presence or absence of potential ligands was investigated by TSA using the Tycho NT.6 (NanoTemper Technologies, Munich, Germany), according to the user manual. Subsequently, 10 µl of samples containing 10 µM of 41O1_SusD-like in Tris buffer (20 mM Tris-HCl, 150 mM NaCl, pH 7.4), with or without ligands, at a final concentration of 0.5% (w/v) was used to fill the capillary tubes. The intrinsic protein fluorescence was recorded at 330 nm and 350 nm while the sample was heated from 35°C to 95°C over a 3-min period.

### NMR spectroscopy

The interaction of 41O1_SusD-like with ligands was assayed by Saturation transfer difference NMR (STD NMR) spectroscopy. All experiments were performed at 298K on a Bruker Avance II 800 MHz NMR spectrometer equipped with a QCPI cryogenically cooled probe head. The samples contained 50 µM 41O1_SusD-like protein with or without ligands (0.1 mM, 1 mM) in a Tris buffer (Tris 20 mM, NaCl 150 mM at pH 7.4).

1D proton spectra were acquired using Watergate water suppression (75), with 256 scans for 41O1_SusD-like. STD experiments were performed with saturation of the protein resonances at 0 ppm through a train of 5 ms Gaussian 180° pulses. Typical experiments were run with 256 scans, 2,048 acquisition points and a 5-s relaxation delay (including the 3-s pre-saturation train). STD experiments were run with 256 or 512 scans. Spectra were transformed after one level of zero-filling and apodization with a π/3 shifted square sine bell.

### AlphaFold2 model of 41O1_SusD-like

Monomeric structures of 41O1_SusD-like were modeled using LocalColabFold 1.5.2, run on 17 April 2023 (53). Thus, the sequence search was performed using MMseqs (76) on a distant server. The targeted sequence (41O1_SusD-like) as aligned against protein sequences from several databases: (i) Uniref30 (77), (ii) a combination of Big Fantastic Database and MGnify, (iii) PDB70 database, and (iv) ColabFoldDB (53). Localcolabfold parameters were set to the default values, with three prediction recycles and the generation of five models. All 3D models were relaxed with default parameters of AF2 using AMBER Force Field. For structural comparisons, the residue numbering corresponds to the full-lenght protein sequences found in the UniProt database (*Bt*SGBP-A: A0A173VPY3, GM_SusD: A0A1H1MMX0).

## SUPPLEMENTAL MATERIAL

Supplemental Figures msphere.00278-24-s0001.pdf Fig. S1 to S4.

ASM does not own the copyrights to Supplemental Material that may be linked to, or accessed through, an article. The authors have granted ASM a non-exclusive, world-wide license to publish the Supplemental Material files. Please contact the corresponding author directly for reuse.
