# Supplementary material for: Biochemical characterization of a SusD-like protein involved in β-1,3-glucan utilization by an uncultured cow rumen Bacteroides
Source: mSphere. 2024 Jul 16;9(8):e00278-24. doi: 10.1128/msphere.00278-24 (PMC11351036; doi:10.1128/msphere.00278-24)
Supplement: Supplemental Figures — Fig. S1 to S4. [file msphere.00278-24-s0001.pdf]

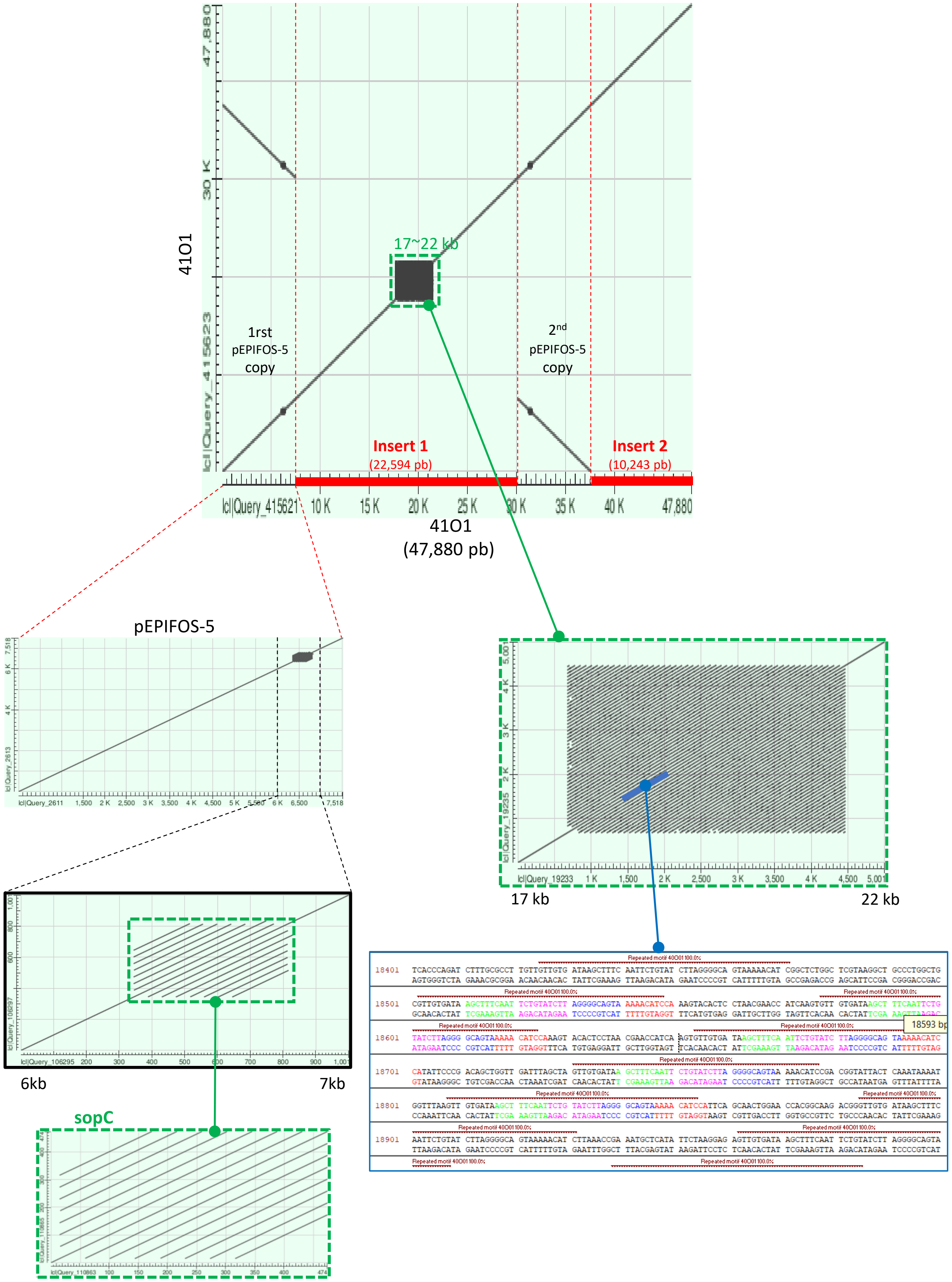

Figure S1: Dot Blot of the 41O1 sequence against itself, using the nucleotide Blastn tool from the NCBI, highlighting repeated sequences in this clone.

The duplication of the pEPIFOS-5 backbone appears as two inverted 7,518 pb sequences, each of them showing the repeated segments within the sopC feature. Another singular feature from this 41O1 clone is the huge, repeated area of almost 4 kb in the middle of the insert 1 sequence, which is made up of the succession of a perfectly conserved 47 pb motif separated by sequences of 20 to 30 pb.

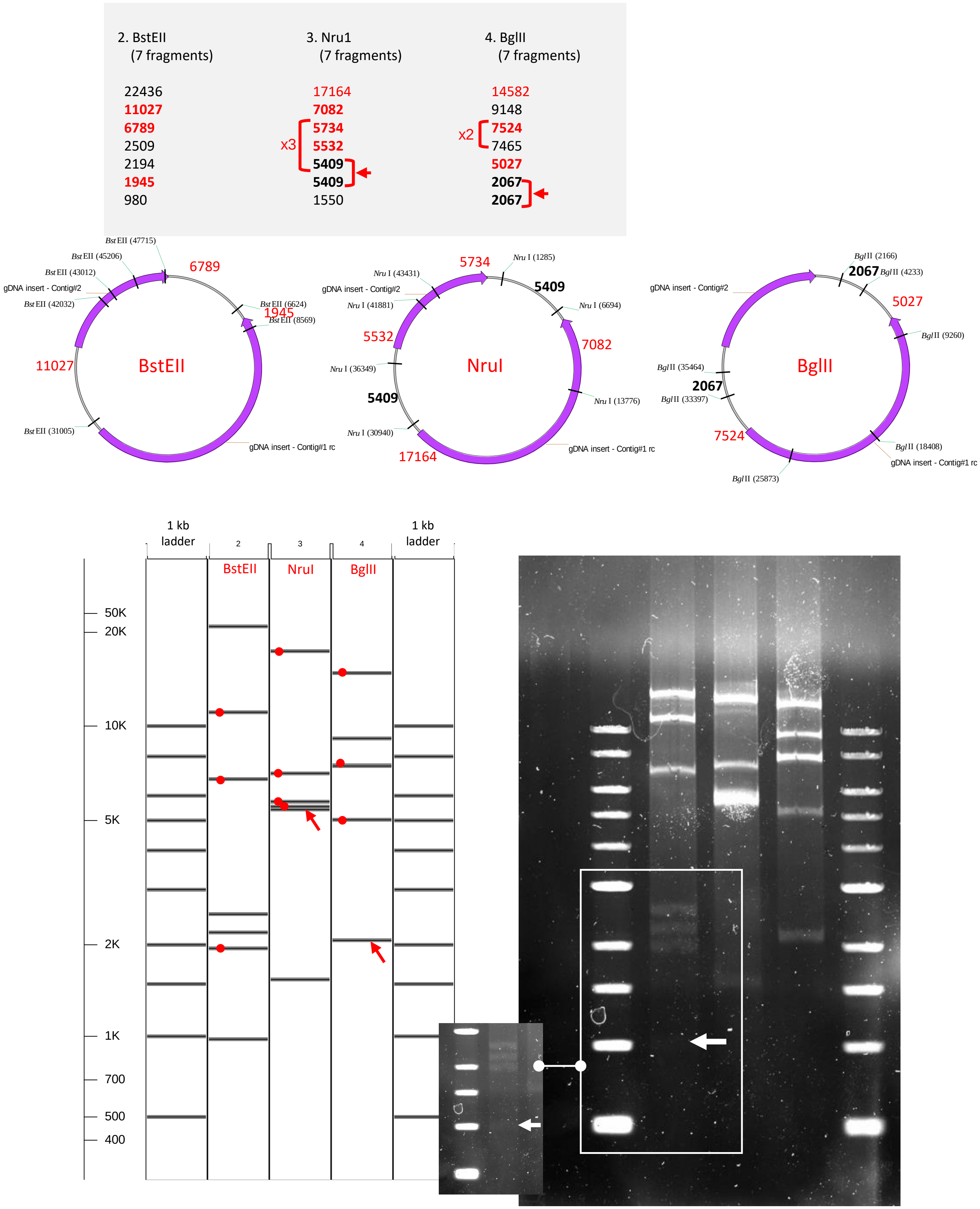

Figure S2: Restriction analysis of the 41O1 clone.
Three restriction enzymes were selected, (i.e. BstE , Nru1, BglII), to provide characteristic restriction patterns highlighting the presence of two independent inserts and backbones. In red, characteristic bands at the backbone/insert junctions. Left : Theoretical migration gel prepared with the VectorNTI software ; Red dots correspond to highlighted, characteristic bands at the junctions. Red arrow points doublets arising from the presence of two backbones (5409 pb and 2067 pb for NruI and BglII restriction , respectively). Right : Agarose migration gel, 0.8 % in TAE. The white arrow targets the 980 pb faint band from BstEII digestion.

A

>41O1_SusD-like
MKRYKNNIVAGFMASALLLTGCSESFLEVESMTQETIDTYFTTDEHIQEAVVAAYDPLHWSDWGIGQYNPL
CIMSDIMADDIWVGGADKNDNQYWHLMMNYEALPTNCMTGLWTVAYSGVKRSNDVLTYIGWAGDNL
TAENATYYEAQARVLRVLYYNWLWKFWGNIPYYEENLTAPYIAEQLSADAVYANIIADLEGAIALDALPMKA
SSSDYGRVTKAMAYMLYAEIVLYQNDESRYAKALAYMQEIINSGQYELVDDYTTIFKESGEWNSESIFEVNYK
DDNAARSWGSPLVAGGTVLPRLISPSGWADGTDGHDNGWGFCPVRLETYERYSNNDARREATCWNAA
AIGSYNTRYQDTGFFLEKYAAKTGDNADQIADADLNYNNNLRIYRFSETLLNAAELIVRGGGSGDAKEYLNR
VHKRAGLTIEVAATIDNIIEERHLEFVGEGKRYWDLIRTGKAASVLVPDSYGYRTNAWTENKKYLPIPQSEIDA
AQGTLTQNNY

B

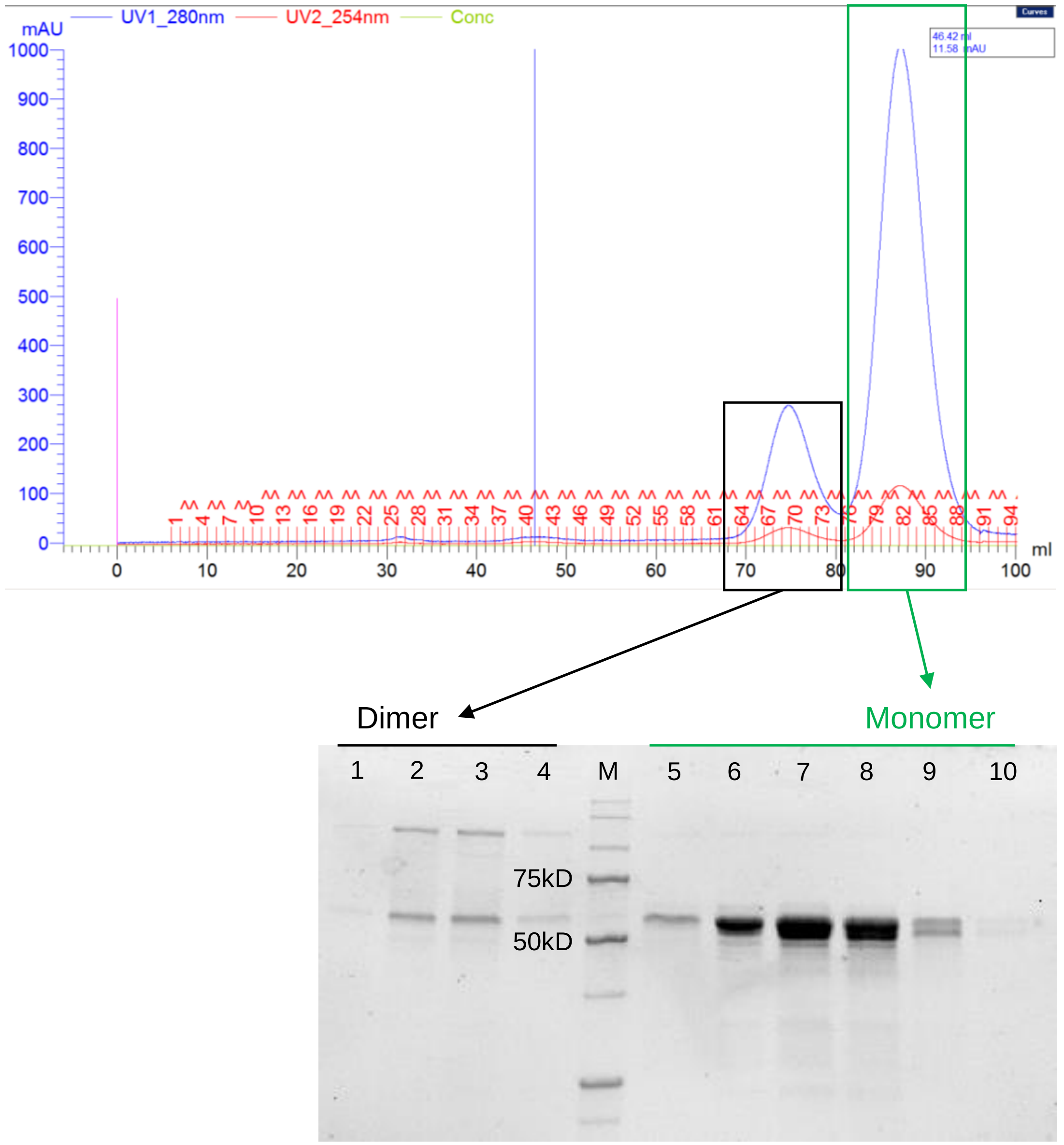

Figure S3: (A) 41O1_SusD-like full-length protein sequence. (B) Gel filtration of 41O1_SusD-like (57 kDa). The protein was polished by gel filtration using an AKTA System (HiPrep 16/60 Sephacryl S-200 HR) (GE-Healthcare, Uppsala, Sweden) in 20 mM Tris - HCl, 150 mM NaCl, pH 7.4. SDS-PAGE gel photographed with BioRad GS-800 calibrated densitometer system. Line M: Precision Plus Protein Unstained Standards marker; Line 1-4: Fractions of dimeric 41O1_SusD-like; Line 5-10: Fractions of monomeric 41O1_SusD-like.

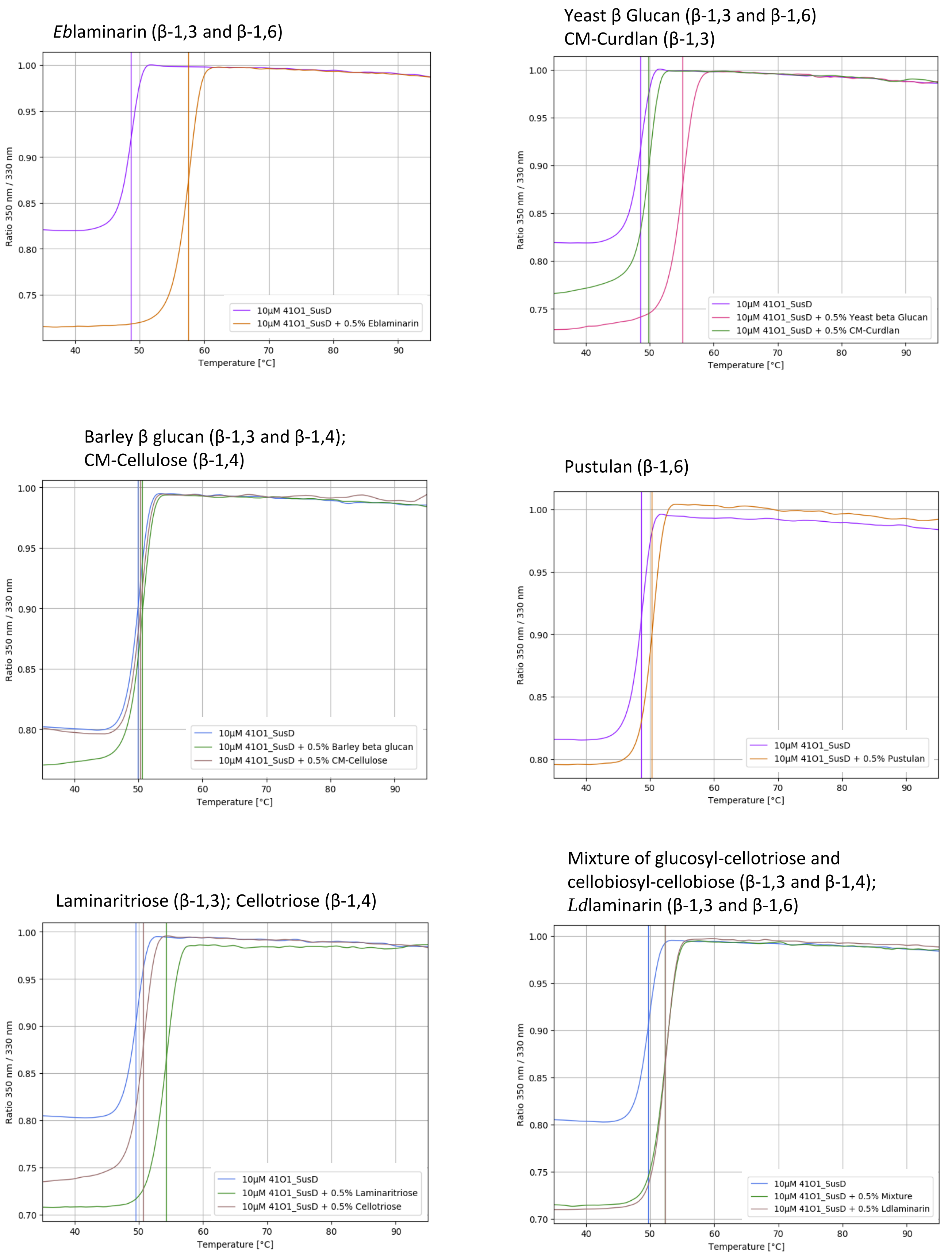

Figure S4: Thermal shift analysis of 10 µM 41O1_SusD-like protein incubated with 0.5% (w/v) ligands. Vertical lines indicate inflection temperatures (**Ti**).
